# Editorial: Sensing the World Through Predictions and Errors

**DOI:** 10.3389/fnhum.2022.899529

**Published:** 2022-04-21

**Authors:** Ryszard Auksztulewicz, Marta I. Garrido, Manuel S. Malmierca, Alessandro Tavano, Juanita Todd, István Winkler

**Affiliations:** ^1^Department of Neuroscience, City University of Hong Kong, Kowloon, Hong Kong SAR, China; ^2^European Neuroscience Institute Göttingen, University Medical Center Göttingen and the Max Planck Society, Göttingen, Germany; ^3^Melbourne School of Psychological Sciences, The University of Melbourne, Melbourne, VIC, Australia; ^4^Cognitive and Auditory Neuroscience Laboratory (CANELAB), Institute for Neuroscience of Castilla y León (INCYL), University of Salamanca, Salamanca, Spain; ^5^Department of Cell Biology and Pathology, The Faculty of Medicine, University of Salamanca, Salamanca, Spain; ^6^The Salamanca Institute of Biomedical Research, Salamanca, Spain; ^7^Department of Neuroscience, Max Planck Institute for Empirical Aesthetics, Frankfurt, Germany; ^8^School of Psychology, The University of Newcastle, Callaghan, NSW, Australia; ^9^Institute of Cognitive Neuroscience and Psychology, Research Centre for Natural Sciences, Budapest, Hungary

**Keywords:** perception, sensory attenuation, prediction error, predictive coding, computational modeling, depression, altered state of consciousness, aging

One of the critical functions of the brain is to prepare for future states and events. Over the past 40 years, several new theories and mounting empirical evidence have emerged in support of predictive information processing in the brain. Arguably, one the most popular theories is that the brain's computational goal is to minimize prediction errors—the difference between predictions and actual sensory inputs. Thus, “errors” are inseparable from prediction itself. In fact, evidence for predictive processing often comes from measuring prediction errors, which reflect sensory deviance detection (with or without awareness). The current Research Topic pulls together theoretical, empirical, and modeling studies on the role of prediction in perception, often tested by how deviation from what is predictable is processed in the brain. As a teaser for potential readers of this Research Topic, we shortly summarize each paper and their wealth of results, from measuring the response to simple forms of sensory deviation, through testing features of the putative predictive coding framework, to assessing how predictive processes of perception operate in different states of the organism, aging, clinical groups, and in conjunction with behavior.

Prediction error signaling is most commonly studied in oddball paradigms, in which an occasional presentation of an unexpected stimulus, deviating from a sequence of expected standard stimuli, evokes a mismatch response. Such unexpected deviant stimuli can differ from the standards based on multiple sensory features. In an electroencephalography (EEG) based study, An et al. tested whether mismatch responses depend on the sensory features constituting auditory deviants. The study manipulated four acoustic features and identified robust mismatch responses which, in a univariate analysis, were indistinguishable across features. However, the features could be decoded from response topography in a multivariate manner, although at relatively late latencies. These results suggest that mismatch detection may occur prior to deviant feature processing. In a magnetoencephalographic study, Xu et al. focused on the somatosensory modality and manipulated deviant stimuli such that they could be unpredictable (replacing a randomly selected standard) or predictable (presented directly after the unpredictable stimuli). The study identified an early activity component that differentiated between unpredictable and predictable deviants, implying its role in prediction error signaling. In contrast, a later activity component differentiated between the deviants and standards, but not between unpredictable and predictable rare stimuli, suggesting that it reflects rareness-related signaling rather than prediction error signaling. Using direct recordings from the cortical surface in rodents, Shiramatsu et al. investigated the relationship between mismatch responses and multisensory integration. Deviant stimuli could be presented in either the auditory or visual modality alone or accompanied by congruent/incongruent stimulation in the other modality. A comparison of mismatch responses across conditions revealed a non-linear relationship between single-modal and cross-modal mismatch responses. Furthermore, local blockage of N-methyl-D-aspartate receptors in the visual cortex diminished mismatch responses to single-modal visual deviants as well as to congruent cross-modal deviants, suggesting cross-modal influences on mismatch signaling.

Going beyond classical oddball paradigm, Kimura investigated visual predictive processing in the context of a phenomenon called representational momentum, which corresponds to a predictive perceptual displacement of a position or rotation of a visual stimulus along its recent regular pattern. By quantifying the amplitude of EEG-based visual event-related potentials (vERP) to a regularly rotated visual bar, the study established an across-participant correlation between the vERP amplitude and subsequent behavioral representational momentum, stressing the role of individual differences in the neural and behavioral correlates of predictive processing.

A corollary of the predictive coding framework is that the saliency of an improbable event increases with the precision of the predictive model, which in turn depends on the variability of the regular features of sound sequences. Increasing the variability of the acoustic regularity reduces the predictive strength of one's internal generative models about the auditory environment. This, in turn is expected to lead to lower precision of predictions and thus a reduced prediction error, indexed by smaller mismatch negativity (MMN) amplitude. Three studies within the current Research Topic of articles tested this hypothesis. SanMiguel et al. demonstrated this empirically by varying regularity stability with ramping the probability of the standard tone and assessing the ERP elicited by the deviant tone. They showed that for the same deviant probability, the MMN amplitude is greater when the probability of the standard increases (i.e., regularity variability decreased). Brace and Sussman found that when two auditory features carry separate regularities, predictions are created for both, irrespective of whether either or none are attended/task relevant. Bader et al. increased the variability of the regularity by replacing one tone within a six-tone pattern with either a white-noise segment (less precise pattern) or a different pitch tone (even less precise pattern). While MMN was similar across conditions, the P3a ERP component was greater for violations of patterns with less regularity variability (greater model precision). In addition, using trial-by-trial modeling of electrocorticographic (ECoG) data, Lecaignard et al. showed that MMN indeed reflects a precision-weighted prediction error that is time-dependent at electrodes located more posteriorly over the scalp than the main MMN response.

How robust, and the same time how flexible, is prediction error as an index of sensory function integrity? The answer to this fundamental question has proven very difficult to provide, as evidenced by Gilbert et al.s' review on disrupted predictions in Major Depression Disorder as far as both sensory deviance detection and reward processing are concerned. To begin casting that picture, Tivadar et al.s' review the evidence for changes in prediction error responses under altered states of consciousness. While the absence of consciousness (e.g., anesthesia and coma) changes the morphology and reduces the amplitude of responses, deviancy may still be registered by sensory-specific neural circuits, e.g., the core auditory cortex. This is confirmed by the study of Nourski et al., who used intracranial electroencephalography (iEEG) to test patients under wake, sedated, and unresponsive stages of anesthesia induction. Using high gamma activity as a dependent measure, they found that core sensory neural circuits (auditory cortex) reflect the positive interaction of local deviant responses generated by short-term stimulation, and global deviant responses generated when stimulation lasts several seconds. Such interaction is reduced but still measurable in sedated participants.

Another often-tacit assumption is that the magnitude of prediction error response should explain a sizeable portion of variance in a tested function, so that a decay in said function would be indexed by a proportionate reduction in deviance detection processes. Said assumption may be difficult to verify. Neubert et al. studied healthy elderly individuals (60–75 years) by correlating the amplitude of the pre-attentive MMN response to violations of predictable sound sequences, with the ability of participants to ignore the same sound sequences used as a behavioral distractor. The absence of a correlation suggests that predictability extraction does not drive the effect of age on predictability-based sensory inhibition. Similarly, Csizmadia et al. found a discontinuity between visual MMN amplitude and the ability to automatically register age of photographed individuals: only in older adults was the visual MMN sensitive to age changes, suggesting the mediation of a familiarity factor. However, if one widens the clinical applications from decay to resilience and expands the dependent measures from ERPs to prediction error-related movements, such as blinks, as was done by Tavano and Kotz, then the relationship between deviancy and behavior may become strong again and reveal novel ways to compose a more complete picture of extensively studied syndromes such as Parkinson's disease.

In sensory attenuation self-generated sensory input is perceived as less intense than the same stimuli generated externally. In a review of this phenomenon in the auditory modality, Kiepe et al. question the traditional explanations based on motor-based forward models and discuss alternative hypotheses regarding the mechanisms underlying sensory attenuation, such as those based on the predictive coding framework. The review also addresses the challenge of isolating Sensory attenuation from other predictive mechanisms.

Predictive coding appeared to have put to bed the longstanding debate around the role of neuronal adaptation in MMN generation, modeled simply as the result of release from repetition suppression. However, in this Research Topic, an updated adaptation model revives the controversy (May) by showing that recurrent interactions *via* feedforward and feedback short-range connections within the auditory cortex can beautifully simulate MMN to omissions and surprising repetitions, which were critical in ruling out previous hypotheses of adaptation as a plausible mechanism of MMN generation. Hence, physiologically-informed modeling forces the reader to rethink the very implementation of prediction error in the brain.

The wide variety of topics emerging in this article Research Topic demonstrates how deeply the notion of predictive processing permeates current scientific thinking of perception. While even some of the basic assumptions for the role of prediction in perception require further testing, significant advances have been made on mapping out a neural system based on predictive principles. Understanding how these predictive principles are implemented in the brain will have critical implication for our fundamental understanding of altered states of consciousness, as well as neurological and psychiatric conditions.

## Author Contributions

All authors listed have made a substantial, direct, and intellectual contribution to the work and approved it for publication.

## Funding

The financial support to MM was provided by the Spanish Agencia Estatal de Investigación (AEI; PID2019-104570RB-I00), the European Union's Horizon 2020 research and innovation programme (grant agreement No 952378 - BrainTwin), and the Foundation Ramón Areces and the Consejería de Educación de la Junta de Castilla y León (SA252P20). JT was supported by the Australian Research Council (DP200102346). IW was supported by the National Research Development and Innovation Office, Hungary (K132642).

## Conflict of Interest

The authors declare that the research was conducted in the absence of any commercial or financial relationships that could be construed as a potential conflict of interest.

## Publisher's Note

All claims expressed in this article are solely those of the authors and do not necessarily represent those of their affiliated organizations, or those of the publisher, the editors and the reviewers. Any product that may be evaluated in this article, or claim that may be made by its manufacturer, is not guaranteed or endorsed by the publisher.

